# The Neuropeptide Oxytocin Enhances Information Sharing and Group Decision Making Quality

**DOI:** 10.1038/srep40622

**Published:** 2017-01-11

**Authors:** Tim R. W. De Wilde, Femke S. Ten Velden, Carsten K. W. De Dreu

**Affiliations:** 1 Department of Psychology, University of Amsterdam, P.O. Box 15919, 1001 NK Amsterdam, The Netherlands; 2 Institute of Psychology, Leiden University, P.O. Box 9555, 2300 RB Leiden, The Netherlands; 3 Center for Experimental Economics and Political Decision Making, University of Amsterdam, P.O. Box 15551, 1001 NB Amsterdam, Netherlands.

## Abstract

Groups can make better decisions than individuals when members cooperatively exchange and integrate their uniquely held information and insights. However, under conformity pressures group members are biased towards exchanging commonly known information, and away from exchanging unique information, thus undermining group decision-making quality. At the neurobiological level, conformity associates with the neuropeptide oxytocin. A double-blind placebo controlled study found no evidence for oxytocin induced conformity. Compared to placebo groups, three-person groups whose members received intranasal oxytocin, focused more on unique information (i) and repeated this information more often (ii). These findings reveal oxytocin as a neurobiological driver of group decision-making processes.

Whether in politics, governance or industry, decision-making is often delegated to groups. These groups make important decisions that impact the lives of many. Groups have the potential of making decisions of higher quality than individual decision makers when group members open-mindedly contribute and evaluate their own and their fellow group members’ unique information and insights, and when they constructively discuss doubts, criticisms, and competing scenarios^1^,^2^,^3^,^4^,^5^. Conversely, the quality of group decision-making is often threatened by individual self-censorship along with conformity pressures and excessive need to affiliate with others^3^,^4^,^5^,^6^. Such “groupthink”^6^ biases individuals away from sharing uniquely held insights and information, leaving the potential for groups to outperform individuals unrealized. Indeed, groupthink has been connected to the Challenger and Columbia Space Shuttle disasters, the collapse of Enron in 2001, and the recent financial crisis^7^.

Although the socio-motivational antecedents to group information sharing and decision-making are well-understood^1^,^2^,^4^,^8^, its neurobiological underpinnings are sparsely addressed^9^. It stands to reason, however, that critical group decision-making processes and outcomes link to oxytocin, a nine amino-acid hormone and neurotransmitter that is produced in the hypothalamus^10^,^11^,^12^.

Oxytocin has been hypothesized to raise the salience of social cues in general^13^ and to shift individuals’ focus from self- to group-interests in particular^14^. In agreement with these theories, oxytocin facilitates interpersonal coordination^15^; increases positive communication between couples^16^ and raises cooperation towards the in-group in social dilemma settings^17^. Yet, these processes do not always result in positive outcomes, as indicated by work showing that oxytocin increases dishonesty to favor one’s group^18^, envy, and gloating^19^.

Effects of oxytocin induced group-focus might also not be unequivocally beneficial for group decision-making, but rather depend on the magnitude and scope of such group focus. As stated above, group decision quality tends to benefit from open-minded exchange of ideas and information, during which the quality of contributions is critically processed and monitored. For this to occur group-focus should be moderate. When group-focus is absent and group members are mostly focused on their personal interests, decision quality tends to decrease due to unwillingness to exchange information^20^ or due to a tendency to distort information^21^. In contrast, when group members are too much focused on their group, they might resort to conformity and shallow compromising by negotiating preferences instead of discussing information, which also decreases group performance^5^,^6^,^22^.

There is some evidence that oxytocin indeed results in such conformity driven decision-making: Under oxytocin people are more likely to conform to their group members on factual^23^, and judgmental issues^24^,^25^. This willingness to align one’s opinion to others – based on little more than joined group membership – suggests that oxytocin might reduce group decision-quality by resorting to quick compromises in which members with a minority preference conform to those members with the majority preference. This type of decision-making process undermines the decision-quality further because information exchange tends to be limited in such preference-driven discussions^8^,^22^.

A recent study, however, indicates that oxytocin induced conformity might not be based on simple blind followership but on the careful monitoring of one’s own and other members’ capabilities. Hertz and colleagues^26^ showed that dyads working on a visual search task performed better when given intranasal oxytocin because the less performing group members were more willing to conform to the opinion of the best members, while the best members became less willing to conform to their worse performing partners. Such an effect of oxytocin would be beneficial because adopting views of people with new and useful information increases (group) decision-quality^27^.

Whether and how oxytocin influences decision making processes and quality in group decision making is examined in a double-blind placebo controlled experiment in which three-person groups perform a hidden profile task, a well-established group decision making assignment (Fig. 1; *Methods*). In this task, some information is shared by all group members and some information is unique for each group member. In order to perform well, group members should exchange all information – with unique information being crucial for identifying the correct alternative. Participants individually read case materials about a murder they had to solve that included, among other things, interviews with three suspects^28^. Next, participants indicated their individual preference for one of the suspects and discussed the case with their two group members using a chat-program. Chats were coded for case-relevant indicators of information exchange and repetition (*Methods*), and group decisions were recorded as “incorrect” or “correct”.

## Results

Treatment was unrelated to age, *t*(112) = −0.17, *p* = 0.863, gender χ^2^(1, *N* = 114) = 0.15, *p* = 0.702, and gender composition of the groups, χ^2^(3, *N* = 38) = 1.27, *p* = 0.731. Moreover, gender composition did not relate to any of our dependent variables and is therefore not discussed further.

Treatment was related to the total amount of exchanged information (*M*_OT_ = 16.47, *SD*_OT_ = 8.75; *M*_PL_ = 12.05, *SD*_PL_ = 7.85), however this effect was marginal, *t*(36) = −1.77, *p* = 0.085. This effect on the total amount of exchanged information was due to the exchange of unique information (*M*_OT_ = 7.89, *SD*_OT_ = 4.62; *M*_PL_ = 5.05, *SD*_PL_ = 4.82), *t*(36) = −2.35, *p* = 0.025, and not the exchange of shared information (*M*_OT_ = 8.58, *SD*_OT_ = 5.77; *M*_PL_ = 7.00, *SD*_PL_ = 3.76), *t*(36) = −0.80, *p* = 0.428.

More interestingly, oxytocin significantly increased the proportion of the discussion groups spent on exchanging unique information. A 2 (proportion of unique/shared information) × 2 (treatment) mixed-model Analysis of Variance (ANOVA) revealed that, overall, shared information was discussed more (*M* = 0.58, *SD* = 0.21) than unique information (*M* = 0.42, *SD* = 0.21), *F*(1, 36) = 6.44, *p* = 0.016, η_p_^2^ = 0.152. The proportion of unique and shared information depended on treatment, such that groups receiving oxytocin (*M* = 0.49, *SD* = 0.18) shared more unique information (and thus less shared information) than groups given a placebo (*M* = 0.34, *SD* = 0.22), *F*(1, 36) = 5.29, *p* = 0.027, η_p_^2^ = 0.128 (see Fig. 2A; given that the two proportions are each other’s inverse, this results equals a direct comparison of conditions in a One-Way ANOVA). The bias in favor of shared information was only present in groups given placebo, *F*(1, 36) = 11.70, *p* = 0.002, and absent in groups given oxytocin, *F*(1, 36) = 0.03, *p* = 0.867. Moreover, the proportion of unique information in oxytocin groups exceeded what one would predict based on the distribution of shared (15; 62.5%) and unique items (9; 37.5%), *t*(18) = 2.85, *p* = 0.011, which was not the case for placebo groups, *t*(18) = −0.66, *p* = 0.518.

In addition to information exchange, oxytocin also influenced the processing of information during group discussions, as evidenced by repetition scores. A Mann-Whitney test indicated that repetition of unique arguments was higher in oxytocin groups (*Mdn* = 2.00, *M* = 2.12, *SD* = 0.61) than in placebo groups (*Mdn* = 1.33, *M* = 1.36, *SD* = 0.97), *U* = 89.50, *p* = 0.006, *r* = −0.43. However, oxytocin (*Mdn* = 1.40, *M* = 1.47, *SD* = 0.43) and placebo groups (*Mdn* = 1.25, *M* = 1.35, *SD* = 0.36) repeated shared information equally often, *U* = 148.50, *p* = 0.354, *r* = −0.15. Unique information was repeated (as indicated by a repetition score > 1) in only 10 out of 19 placebo groups (53%), and in no less than 18 out of 19 oxytocin groups (95%), χ^2^(1, *N* = 38) = 8.69, *p* = 0.003, *r* = 0.48.

Next, we examined if this increased information processing and exchange of unique information also resulted in better decisions. Prior to group discussion, individuals given oxytocin were equally likely to select the correct alternative (35%) as those given a placebo (32%), χ^2^(1, *N* = 114) = 0.16, *p* = 0.691; both percentages correspond to chance level of selecting the correct alternative (33.33%). Furthermore, in the placebo condition (32%) and the oxytocin condition (26%) there was an approximately equal percentage of groups in which the majority preferred the correct alternative, χ^2^(1, *N* = 38) = 0.13, *p* = 0.721. Following group discussion, 74% of oxytocin groups identified the correct alternative, compared to 47% of placebo groups, χ^2^(1, *N* = 38) = 2.75, *p* = 0.097, Odds Ratio = 3.11 (Fig. 2B). Furthermore, whereas oxytocin groups chose the correct alternative significantly more than would be predicted by chance (33.33%), χ^2^(1, *N* = 19) = 14.22, *p* *<* 0.001*, r* = 0.87, placebo groups did not, χ^2^(1, *N* = 19) = 1.77, *p* = 0.183.

Mediation analysis showed that groups’ focus on unique information predicted superior group decisions. The indirect effect using a bootstrapping method with 10,000 samples showed that oxytocin affected the proportion of unique information which in turn affected decision quality (*B*_*indirect-effect*_ = 1.94, *SE* = 2.25, Bias corrected CI_95_: 0.03–5.61; Figs 2B and 3; ref. 29).

Finally, we investigated signs of conformity. While increased information exchange and processing in itself can be seen as lack of conformity pressure, we also investigated whether group members switched their preferences during the group discussion. Approximately the same amount of group members switched in the placebo (32) and oxytocin (31) groups, however under oxytocin group members made more correct switches (i.e. from the wrong to the correct suspect; 81%) than placebo group members (56%), χ^2^(1, *N* = 63) = 4.33, *p* = 0.038. Moreover, as stated above, prior to group discussion, an equal percentage of groups in both treatments had a two-member majority preferring the correct answer (26% in oxytocin condition). Thus, in three-quarters of the oxytocin groups, conformity pressures should have resulted in incorrect group decisions. However, an overwhelming 74% of all oxytocin groups chose the correct alternative.

## Conclusions and Discussion

When groups received oxytocin this resulted in increased exchange and processing of new information. This finding is important because carefully processing and integrating of new information is essential for reaching high quality decisions^2^,^4^,^5^,^8^,^30^,^31^. Results of our study points in the same direction: After group discussion the solve-rate of oxytocin groups was 26% higher than that of placebo groups. While not reaching statistical significance, this effect on decision quality in combination with significant effects on both information exchange and information processing indicates that decision-making in groups is facilitated by oxytocin. As happens in most decision-making groups^5^, groups whose members received placebo were biased towards the exchange of commonly held information and were less likely to repeat unique information. However, groups whose members received oxytocin had more un-biased discussions and used each other’s inputs better.

These results resonate with recent work showing that oxytocin leads to selective conformity to the best performing member of a team^26^. If, as was suggested by previous work^23^,^24^,^25^, oxytocin stimulates quick “uncritical” conformity to the majority opinion in the group, we should have found that oxytocin groups performed worse after discussion. Instead, our results suggest that oxytocin facilitated the exchange and processing of information, raises members willingness to correctly shift their preference during discussion and thus groups seemed to benefit from oxytocin. In the current task group members needed (and were able) to recognize the correct solution – based on the combination of their and others’ information. Consequently, group members were able to adjust their opinion accordingly when confronted with crucial facts. Therefore, our findings apply to information-based group decision-making, in which a verifiable correct solution is available.

There are cases in which groups have no objectively verifiable solutions and where group decisions are, ultimately, “a matter of taste”^4^,^32^. It cannot be excluded that (oxytocin-induced) pressures towards conformity operate more strongly when group decisions are evaluative and opinion-based and have no identifiable solution (as was the case in the earlier cited work on conformity)^24^,^25^. In addition, such opinion-based situations might elicit more competition among group members, as people tend to identify more strongly with their personal opinions and preferences, than with more factual information given to them^33^,^34^. It would, accordingly, be interesting to examine the role of oxytocin in group-decision making when individuals have stronger motivation to compete than was the case in the currently used more cooperative task^35^.

Present findings resonate with the more general hypotheses about oxytocin shifting individuals’ focus from self- to group-interests^14^. Similar to the task used by Hertz and colleagues^26^, in group decision making the group’s outcome is supported by adopting a goal to perform well as a group. Individualistic motives such as proving competence, gaining status, being faster than others and convincing others would undermine group performance^20^,^35^. In contrast, group performance would benefit from independent thinking (as opposed to conformity). This relates to the finding that oxytocin facilitates creativity^36^, which can be regarded as a specific type of independent, out-of-the-box, thinking. In addition, group decision-making depends on coordination between group members (e.g., who says what and when ? ). Studies have shown that under a common goal, oxytocin facilitates brain responses to social synchrony^37^ and coordinating movements resulting in better performance on a drawing task^15^.

Previous work has shown that trust is a predictor of information exchange^20^,^38^. The current study did not include any data to directly measure trust. Therefore, we cannot exclude the possibility that (part of) our results can be explained by increased trust within groups under oxytocin. Although a recent meta-analysis revealed no overall effect of oxytocin on trust^39^, there is evidence that oxytocin does increase trust among individuals that are part of the same group and have are a common task to perform (For a review see ref. 14). Accordingly, future research should include trust as a potential mediator to explain effects of oxytocin on information exchange in highly interactive groups.

There are several possible neurophysiological pathways through which intranasal oxytocin operates on brain and behavior^40^,^41^. One possibility is that intranasally administered oxytocin passes the blood-brain barrier, and directly targets specific regions and networks in the human brain. Indeed, recent work in humans^41^ and animals^42^,^43^,^44^ shows brain-level increases in oxytocin following intranasal administration. Another, complementing possibility is that intranasal oxytocin reduces HPA-axis activity and lowers cortisol-levels in body and brain (e.g. ref. 16). Detecting which pathway(s) are involved is important, also given the present observation that oxytocin may be a key neurobiological driver of information sharing within groups. One important avenue for new research is to focus on the inter-play between oxytocin and other hormones, such as cortisol. Cortisol can interact with oxytocin^45^ and has been related to (risky) decision-making^46^. We expect such new research to generate novel insights into the neuroendocrine adaptations underlying a range of decisions, including those made in, and by groups.

Previous research on oxytocin’s effects on social behavior has mostly been limited to situations in which participants could not freely communicate^15^,^17^,^23^,^24^,^47^,^48^,^49^. In these studies social behavior was inferred from reactions to preprogrammed responses, scenarios or (patterns of) choices; communication was not possible, not allowed or limited to exchanging choices via the computer^15^,^17^,^23^,^24^,^47^,^48^,^49^. Few studies^16^,^26^ examining the effects of intranasal oxytocin have included unstructured communication and free exchange of information. The current study adds to this work by examining oxytocin in a socially rich context, in which participants met each other before the task and in which they could freely exchange information. In this less controlled yet arguably more realistic setting oxytocin facilitated group decision-making by rendering decision makers more open for others’ information and more willing to share valuable unique information, thereby underlining oxytocin’s role in guiding human social interaction. The current study focused on written, computer-mediated communication without face-to-face contact. Although meta-analytic work has shown that both group decision-making quality and information-exchange does not significantly differ between computer-mediated and face-to-face communication^30^, this does not mean that the role of oxytocin is similar in computer-mediated versus face-to-face communication. Indeed, oxytocin facilitates reading body language, emotion recognition and judging facial-expressions^14^,^50^,^51^, all processes that are more relevant in face-to-face settings, than in computer-mediated interactions. Future research should thus extend our findings to face-to-face settings.

Important decisions may be delegated to groups because of the assumption that “two heads know more than one”^2^,^4^. Crucially, these “heads” need to constructively and open-mindedly exchange and build on each member’s unique information, perspectives, and insights. Here we have shown that oxytocin, without any evidence of conformity pressure, enhances information exchange and processing within groups, suggesting that these critical group processes have tractable neurobiological drivers such as oxytocin.

## Methods

### Participants, exclusion criteria, and ethics

Participants were recruited via an online recruiting system and offered a reward of €20 (approx. $22.5) for participating in the two-hour study on group decision-making. Exclusion criteria were significant medical or psychiatric illnesses, drug or alcohol abuse, smoking more than five cigarettes per day, and for females, any doubts about possible pregnancy. Eligible participants were instructed not to smoke or drink alcohol 24 hours prior to the experiment, and to drink nothing except water two hours before the experiment.

We recruited 123 participants, organized randomly in 41 3-person groups (*N* = 60 in the oxytocin, and *N* = 63 in the placebo condition). Sample size was based on two criteria. First, group-decision making studies using a similar hidden-profile task as used here typically have 12 to 25 groups per condition^30^. Second, earlier work in our laboratory focusing on individual-level effects of oxytocin typically has 40 participants per between-subjects condition e.g. ref. 17. Our stopping rule was further determined by lab availability and availability of placebo/oxytocin nose-sprays. We analyzed data of 38 three-person groups, with 114 individuals (78 females, *M*_*age*_ = 21.63, *SD*_*age*_ = 3.36; 3 groups, 1 in the oxytocin condition, did not engage in any information exchange and were therefore removed).

The experiment was approved by the Ethics Review Board of the University of Amsterdam (file number 2015-WOP-4041), adhered to the Helsinki protocols, and the ethics guidelines from the *American Psychological Association*.

### Substance administration

Participants were randomly assigned to the oxytocin or placebo treatment (double-blind, placebo-controlled design). They self-administered 24 IU oxytocin intranasally (Syntocinon-Spray, Novartis; 3 puffs per nostril) or placebo under experimenter supervision. The only difference between the placebo and treatment was the absence versus presence of the active neuropeptide^17^,^24^,^37^.

### Procedure

**Figure 1:**
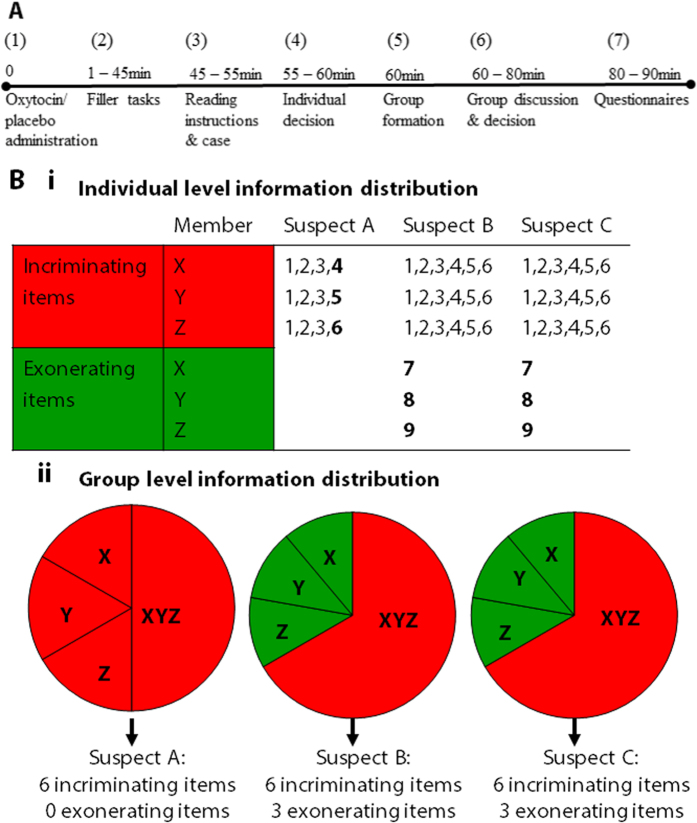
Task and procedure. (**A**) Experimental procedure and timeline. (**B**) Hidden Profile Task Representation. Individual level information distribution (**i**), leading to suspect B or C. Group level information distribution (**ii**) showing, for each suspect, the available information per group member (X, Y, Z). Slices including XYZ represent shared pieces of information; slices including one letter represent unique information. All information combined leads to suspect (**A**).

**Figure 2:**
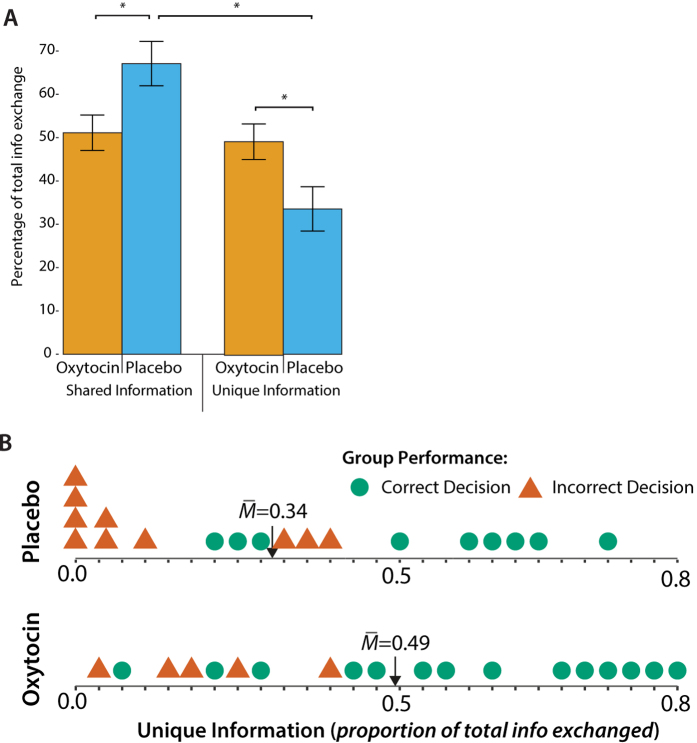
Decision quality and information exchange. (**A**) Unique and shared information as proportion of total information exchange by treatment. Displayed M ± SE. *Significant contrast at *p* < 0.05. (**B**) Unique Information Exchange and Group Performance by Treatment. Circles and triangles show individual groups. Means (M) differ at *p* = 0.014. Unique information predicted decision quality, *B* = 12.77, *SE* = 4.34, *Z*(1) = 2.94, *p* = 0.003.

**Figure 3:**
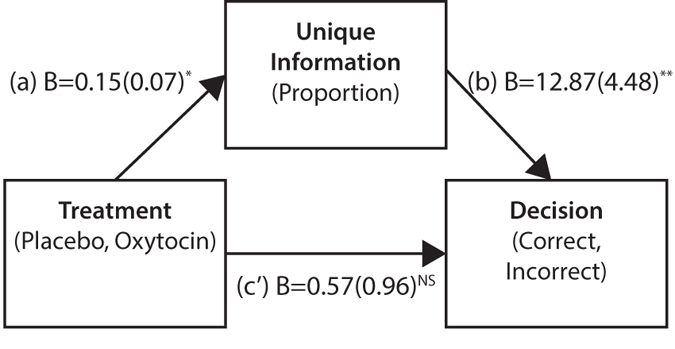
Mediation model using proportion of unique information. SEs are between parentheses. Estimates are based on bootstrapping procedure with 10,000 bootstrap samples. *p < 0.05, **p < 0.01. Used Process Model 4^52^. Path (**a**) *t*(36) = 2.30, *p* = 0.027; Path (**b**) *Z*(1) = 2.87, *p* = 0.004; Path (**c**) *Z*(1) = 0.59, *p* = 0.556.

Upon arrival in the laboratory participants were briefly introduced to each other, and were seated in individual cubicles preventing them from communicating. Participants read and signed the informed consent, and self-administered the medication upon which participants completed a series of unrelated tasks and questionnaires (Fig. 1A).

After 50–60 minutes, instructions appeared for the experimental task for which participants were organized in groups of three. Individuals studied the case materials, and then engaged in the group decision-making task: An instant messenger system popped up on their computer screens, allowing the three group members to communicate by typed chatting. Participants could chat for as long as they wanted, while keeping their materials with them. When the group reached a decision, the experimenter closed the messenger system and re-activated the questionnaire program. Participants responded to several questions, were thanked and dismissed. Participants received their remuneration and debriefing at the end of the study.

## Materials and Measures

### Group decision-making task

We adapted a well-validated “hidden profile task” in which groups of three needed to solve a murder mystery case^28^. Participants were informed that each group member received different information and that they would participate in a computer mediated discussion with the two other people in their group to determine which one of three suspects was guilty (Fig. 1b).

The case description contained 24 relevant arguments that were either incriminating or exonerating for each suspect (Suspect A, B and C). For each suspect there were six incriminating arguments. For both suspect B and C, but not for suspect A, there were also 3 exonerating arguments. Accordingly, when combining all 24 relevant arguments, suspect A was clearly the guilty suspect, while suspects B and C could be ruled out because of the exonerating clues. However, the 24 arguments were not all openly available to each group member (see Fig. 1B). To uncover that suspect A was the guilty suspect, individuals had to exchange and integrate their unique information with their other group members, because shared information falsely led to suspect B or C.

### Information exchange and processing

The discussion transcripts were coded to identify which of the 24 arguments were exchanged by the group. An argument was counted when the essential meaning of the argument was mentioned and attributed to the correct suspect (e.g., “A’s daughter had a row with the victim”). One coder coded all transcripts and a second coder coded 10 transcripts (≈25%). Interrater reliability was excellent, Cohen’s *K* = 0.917, Bias corrected CI_95_: 0.854–0.980, *p* < 0.001^52^.

Coding produced frequency data for each argument. We summarized this data into six variables including the total number of exchanged arguments (1). First, we split up the total arguments into exchanged shared arguments (2) and unique arguments (3). Next we calculated unique arguments as the proportion of the total number of exchanged arguments (4)^28^ in order to investigate to what extent the discussion was focused on either shared or unique information. Finally, we calculated repetition scores as the average number of times shared (5) or unique (6) arguments were mentioned in the group. When zero, no arguments were mentioned, when one, argument(s) were mentioned only once (i.e. no repetition), when two, on average arguments were mentioned twice during the group discussion. The two repetition score variables are treated as ordinal variables since scores between zero and one are not defined in the measure^53^. In line with earlier research, repetition is considered a form of information-processing and is therefore different from simple exchange of arguments^54^,^55^.

### Decision quality

We assessed the quality of decision making, operationalized as “incorrect” (0; choosing suspect B or C) or “correct” (1; choosing suspect A).

### Auxiliary self-report measures

Participants rated certainty with the individual and group decision; agreement with the group decision; ease of making a group decision; the extent the group thoroughly discussed information; whether they focused on fast decision-making and how well they identified with the group. These measures are reported in the online Supplementary Information.

### Analyses

Prior to testing our hypotheses we investigated whether our dependent variables were distributed normally. Deviance from normality was assessed by examining Q-Q plots and by the Shapiro-Wilk’s statistic (*W*) which has most power in assessing deviances from normality^56^. A square-root transformation was used for the total amount of exchanged arguments, *W*_pretransform_ (38) = 0.93, *p* = 0.021; *W*_posttransform_ (38) = 0.97, *p* = 0.387; and the amount of shared, *W*_pretransform_ (38) = 0.90, *p* = 0.002; *W*_posttransform_ (38) = 0.97, *p* = 0.311 and unique arguments, *W*_pretransform_ (38) = 0.93, *p* = 0.023; *W*_posttransform_ (38) = 0.95, *p* = 0.068. The proportion of unique arguments was not transformed, *W*(38) = 0.96, *p* = 0.144. All reported Means and SD’s are untransformed. Repetition scores were analyzed with non-parametric statistics given the ordinal nature of this data.

## Additional Information

**How to cite this article**: Wilde, T. R. W. *et al*. The Neuropeptide Oxytocin Enhances Information Sharing and Group Decision Making Quality. *Sci. Rep.* **7**, 40622; doi: 10.1038/srep40622 (2017).

**Publisher's note:** Springer Nature remains neutral with regard to jurisdictional claims in published maps and institutional affiliations.

## Supplementary Material

Supporting Online Material

## References

[b1] Hinsz V. B., Tindale R. S. & Vollrath D. a. The emerging conceptualization of groups as information processors. Psychol. Bull. 121, 43–64 (1997). 9000891 10.1037/0033-2909.121.1.43

[b2] Kerr N. L. & Tindale R. S. Group performance and decision making. Annu. Rev. Psychol. 55, 623–655 (2004). 14744229 10.1146/annurev.psych.55.090902.142009

[b3] Kruglanski A. W., Pierro A., Mannetti L. & De Grada E. Groups as epistemic providers: Need for closure and the unfolding of group-centrism. Psychol. Rev. 113, 84–100 (2006). 16478302 10.1037/0033-295X.113.1.84

[b4] De Dreu C. K. W., Nijstad B. A. & Van Knippenberg D. Motivated information processing in group judgment and decision making. Personal. Soc. Psychol. Rev. 12, 22–49 (2008). 10.1177/1088868307304092 18453471

[b5] Schulz-Hardt S. & Mojzisch A. How to achieve synergy in group decision making: Lessons to be learned from the hidden profile paradigm. Eur. Rev. Soc. 23, 305–343 (2012).

[b6] Janis I. L. & Mann L. Decision making: A psychological analysis of conflict, choice and commitment. (Free Press, 1977).

[b7] Bénabou R. Groupthink: Collective delusions in organizations and markets. Rev. Econ. Stud. 80, 429–462 (2013).

[b8] Schulz-Hardt S., Brodbeck F. C., Mojzisch A., Kerschreiter R. & Frey D. Group decision making in hidden profile situations: dissent as a facilitator for decision quality. J. Pers. Soc. Psychol. 91, 1080–93 (2006). 17144766 10.1037/0022-3514.91.6.1080

[b9] Faber N. S., Häusser J. A. & Kerr N. L. Sleep deprivation impairs and caffeine enhances my performance, but not always our performance: How acting in a group can change the effects of impairments and enhancements. Personal. Soc. Psychol. Rev, doi: 10.1177/1088868315609487 (2015). PMC5302073 26468077

[b10] Carter C. S. Oxytocin pathways and the evolution of human behavior. Annu. Rev. Psychol. 65, 17–39 (2014). 24050183 10.1146/annurev-psych-010213-115110

[b11] Rilling J. K. & Young L. J. The biology of mammalian parenting and its effect on offspring social development. Science (80-.). 345, 771–776 (2014). 10.1126/science.1252723 PMC4306567 25124431

[b12] Meyer-Lindenberg A., Domes G., Kirsch P. & Heinrichs M. Oxytocin and vasopressin in the human brain: Social neuropeptides for translational medicine. Nat. Rev. Neurosci. 12, 524–538 (2011). 21852800 10.1038/nrn3044

[b13] Shamay-Tsoory S. G. & Abu-Akel A. The social salience hypothesis of oxytocin. Biol. Psychiatry 79, 1–9 (2015). 10.1016/j.biopsych.2015.07.020 26321019

[b14] De Dreu C. K. W. & Kret M. E. Oxytocin conditions intergroup relations through upregulated in-group empathy, cooperation, conformity, and defense. Biol. Psychiatry 79, 165–173 (2016). 25908497 10.1016/j.biopsych.2015.03.020

[b15] Arueti M. . When two become one: The role of oxytocin in interpersonal coordination and cooperation. J. Cogn. Neurosci. 25, 1418–1427 (2013). 23574582 10.1162/jocn_a_00400

[b16] Ditzen B. . Intranasal oxytocin increases positive communication and reduces cortisol levels during couple conflict. Biol. Psychiatry 65, 728–731 (2009). 19027101 10.1016/j.biopsych.2008.10.011

[b17] De Dreu C. K. W. . The neuropeptide oxytocin regulates parochial altruism in intergroup conflict among humans. Science (80-.). 328, 1408–11 (2010). 10.1126/science.1189047 20538951

[b18] Shalvi S. & De Dreu C. K. W. Oxytocin promotes group-serving dishonesty. Proc. Natl. Acad. Sci. 111, 5503–5507 (2014). 24706799 10.1073/pnas.1400724111 PMC3992689

[b19] Shamay-Tsoory S. G. . Intranasal administration of oxytocin increases envy and schadenfreude (gloating). Biol. Psychiatry 66, 864–870 (2009). 19640508 10.1016/j.biopsych.2009.06.009

[b20] Toma C. & Butera F. Hidden profiles and concealed information: strategic information sharing and use in group decision making. Pers. Soc. Psychol. Bull. 35, 793–806 (2009). 19332434 10.1177/0146167209333176

[b21] Steinel W., Utz S. & Koning L. The good, the bad and the ugly thing to do when sharing information: Revealing, concealing and lying depend on social motivation, distribution and importance of information. Organ. Behav. Hum. Decis. Process. 113, 85–96 (2010).

[b22] Brodbeck F. C., Kerschreiter R., Mojzisch A. & Schulz-Hardt S. Asymmetries model under group decision making knowledge: Conditions of distributed model the information asymmetries. Acad. Manag. Rev. 32, 459–479 (2014).

[b23] Edelson M. G. . Opposing effects of oxytocin on overt compliance and lasting changes to memory. Neuropsychopharmacology 40, 966–973 (2015). 25308350 10.1038/npp.2014.273 PMC4330510

[b24] Stallen M., De Dreu C. K. W., Shalvi S., Smidts A. & Sanfey A. G. The herding hormone: Oxytocin stimulates in-group conformity. Psychol. Sci. 23, 1288–1292 (2012). 22991128 10.1177/0956797612446026

[b25] Huang Y., Kendrick K. M., Zheng H. & Yu R. Oxytocin enhances implicit social conformity to both in-group and out-group opinions. Psychoneuroendocrinology 60, 114–119 (2015). 26143536 10.1016/j.psyneuen.2015.06.003

[b26] Hertz U. . Oxytocin effect on collective decision making: A randomized placebo controlled study. PLoS One 11, 1–16 (2016). 10.1371/journal.pone.0153352 PMC4829266 27070542

[b27] Bonaccio S. & Dalal R. S. Advice taking and decision-making: An integrative literature review, and implications for the organizational sciences. Organ. Behav. Hum. Decis. Process. 101, 127–151 (2006).

[b28] Stasser G. & Stewart D. Discovery of hidden profiles by decision-making groups: Solving a problem versus making a judgment. J. Pers. Soc. Psychol. 63, 426–434 (1992).

[b29] Hayes A. F. Introduction to mediation, moderation, and conditional process analysis: A regression-based approach. (Guilford Press, 2013).

[b30] Lu L., Yuan Y. C. & McLeod P. L. Twenty-five years of hidden profiles in group decision making: a meta-analysis. Personal. Soc. Psychol. Rev. 16, 54–75 (2012). 10.1177/1088868311417243 21896790

[b31] Stasser G. & Titus W. Pooling of unshared information in group decision making: Biased information sampling during discussion. J. Personal. Soc. Psychol. 48, 1467–1478 (1985).

[b32] Kaplan M. F. & Miller C. E. Group decision making and normative versus informational influence: Effects of type of issue and assigned decision rule. J. Pers. Soc. Psychol. 53, 306–313 (1987).

[b33] De Dreu C. K. W. & Van Knippenberg D. The possessive self as a barrier to conflict resolution: effects of mere ownership, process accountability, and self-concept clarity on competitive cognitions and behavior. J. Pers. Soc. Psychol. 89, 345–57 (2005). 16248718 10.1037/0022-3514.89.3.345

[b34] Pomerantz E. M., Chaiken S. & Tordesillas R. S. Attitude strength and resistance processes. J. Pers. Soc. Psychol. 69, 408–19 (1995). 7562388 10.1037//0022-3514.69.3.408

[b35] Wittenbaum G. M., Hollingshead A. B. & Botero I. C. From cooperative to motivated information sharing in groups: moving beyond the hidden profile paradigm. Commun. Monogr. 71, 286–310 (2004).

[b36] De Dreu C. K. W. . Oxytonergic circuitry sustains and enables creative cognition in humans. Soc. Cogn. Affect. Neurosci. 9, 1159–1165 (2014). 23863476 10.1093/scan/nst094 PMC4127019

[b37] Levy J. . Oxytocin selectively modulates brain response to stimuli probing social synchrony. Neuroimage 124, 923–930 (2016). 26455794 10.1016/j.neuroimage.2015.09.066

[b38] Butler J. K. J. Trust expectations, information sharing, climate of trust, and negotiation effectiveness and efficiency. Gr. Organ. Manag. 24, 217–238 (1999).

[b39] Nave G., Camerer C. & McCullough M. Does oxytocin increase trust in humans? A critical review of research. Perspect. Psychol. Sci. 10, 772–789 (2015). 26581735 10.1177/1745691615600138

[b40] Leng G. & Ludwig M. Intranasal oxytocin: Myths and delusions. Biol. Psychiatry 1–8, doi: 10.1016/j.biopsych.2015.05.003 (2015). 26049207

[b41] Paloyelis Y. . A Spatiotemporal Profile of *In Vivo* Cerebral Blood Flow Changes Following Intranasal Oxytocin in Humans. Biol. Psychiatry 79, 693–705 (2016). 25499958 10.1016/j.biopsych.2014.10.005

[b42] Dal Monte O., Noble P. L., Turchi J., Cummins A. & Averbeck B. B. CSF and blood oxytocin concentration changes following intranasal delivery in macaque. PLoS One 9, e103677 (2014). 25133536 10.1371/journal.pone.0103677 PMC4136720

[b43] Modi M. E., Connor-Stroud F., Landgraf R., Young L. J. & Parr L. A. Aerosolized oxytocin increases cerebrospinal fluid oxytocin in rhesus macaques. Psychoneuroendocrinology 45, 49–57 (2014). 24845176 10.1016/j.psyneuen.2014.02.011 PMC4120060

[b44] Neumann I. D., Maloumby R., Beiderbeck D. I., Lukas M. & Landgraf R. Increased brain and plasma oxytocin after nasal and peripheral administration in rats and mice. Psychoneuroendocrinology 38, 1985–1993 (2013). 23579082 10.1016/j.psyneuen.2013.03.003

[b45] Heinrichs M., Baumgartner T., Kirschbaum C. & Ehlert U. Social support and oxytocin interact to suppress cortisol and subjective responses to psychosocial stress. Biol. Psychiatry 54, 1389–1398 (2003). 14675803 10.1016/s0006-3223(03)00465-7

[b46] van den Bos R., Harteveld M. & Stoop H. Stress and decision-making in humans: Performance is related to cortisol reactivity, albeit differently in men and women. Psychoneuroendocrinology 34, 1449–1458 (2009). 19497677 10.1016/j.psyneuen.2009.04.016

[b47] Kosfeld M., Heinrichs M., Zak P. J., Fischbacher U. & Fehr E. Oxytocin increases trust in humans. Nature 435, 673–676 (2005). 15931222 10.1038/nature03701

[b48] Baumgartner T., Heinrichs M., Vonlanthen A., Fischbacher U. & Fehr E. Oxytocin Shapes the Neural Circuitry of Trust and Trust Adaptation in Humans. Neuron 58, 639–650 (2008). 18498743 10.1016/j.neuron.2008.04.009

[b49] Rilling J. K. . Effects of intranasal oxytocin and vasopressin on cooperative behavior and associated brain activity in men. Psychoneuroendocrinology 37, 447–461 (2012). 21840129 10.1016/j.psyneuen.2011.07.013 PMC3251702

[b50] Bartz J. A., Zaki J., Bolger N. & Ochsner K. N. Social effects of oxytocin in humans: context and person matter. Trends Cogn. Sci. 15, 301–309 (2011). 21696997 10.1016/j.tics.2011.05.002

[b51] Kemp A. H. & Guastella A. J. The role of oxytocin in human affect: A novel hypothesis. Curr. Dir. Psychol. Sci. 20, 222–231 (2011).

[b52] Landis J. R. & Koch G. G. The measurement of observer agreement for categorical data. Biometrics 33, 159–174 (1977). 843571

[b53] Field A. Discovering statistics using IBM SPSS statistics. (Sage, 2013).

[b54] Scholten L., van Knippenberg D., Nijstad B. A. & De Dreu C. K. W. Motivated information processing and group decision-making: Effects of process accountability on information processing and decision quality. J. Exp. Soc. Psychol. 43, 539–552 (2007).

[b55] Larson J. R., Christensen C., Franz T. M. & Abbott A. S. Diagnosing groups: the pooling, management, and impact of shared and unshared case information in team-based medical decision making. J. Pers. Soc. Psychol. 75, 93–108 (1998). 9686452 10.1037//0022-3514.75.1.93

[b56] Razali N. M. & Wah Y. B. Power comparisons of shapiro-wilk, kolmogorov-smirnov, lilliefors and anderson-darling tests. J. Stat. Model. Anal. 2, 21–33 (2011).

