# Supplementary material for: The Neuropeptide Oxytocin Enhances Information Sharing and Group Decision Making Quality
Source: Sci Rep. 2017 Jan 11;7:40622. doi: 10.1038/srep40622 (PMC5225413; doi:10.1038/srep40622)
Supplement: Supporting Online Material [file srep40622-s1.pdf]

Supporting Online Material for

The Neuropeptide Oxytocin Enhances Information Sharing and Group Decision Making Quality

Tim R.W. de Wilde[a,*], Femke S. Ten Velden[a] & Carsten K.W. De Dreu[b,c]

[a]Department of Psychology, University of Amsterdam

[b]Institute of Psychology, Leiden University

[c]Center for Experimental Economics and Political Decision Making, University of Amsterdam

[*]Corresponding Author: University of Amsterdam, PO Box 15919

1001 NK Amsterdam, The Netherlands. Email: t.r.w.dewilde@uva.nl

Additional Information on Methods and Results

Discussion time and speaking turns

Discussion time was measured by the computer, and the number of speaking turns were counted from the discussion transcripts. Treatment did not influence discussion time (M_{OT}=21.01 minutes, SD_{OT}=12.50; M_{PL}=17.61, SD_{PL}=10.51), $t(36)=–0.91$, $p=.369$, the number of speaking turns (M_{OT}=80.89, SD_{OT}=39.52; M_{PL}=69.47, SD_{PL}=41.50), $t(36)=–0.87$, $p=.391$.

Certainty, decision ease and agreement

Certainty (1=*very uncertain;* 7=*very certain*) was measured both before (relating to the individual decision participants made) and after group discussion (relating to the group's decision). In the post-task questionnaires participants also rated their agreement with the group decision (1=*strongly disagree;* 7=*strongly agree*) and how easy it was to reach a group decision (1=*very difficult;* 7=*very easy*).

Whereas intranasal administration of oxytocin enabled groups to better exchange and process information, this increase in quality was not reflected in post-discussion agreement with the group’s decision, $t(36)=0.74$, $p=.467$, or perceived ease of decision-making, $t(36)=1.18$, $p=.245$ (for Means and Standard Deviations, see Table S1). Finally, a 2(pre/post-discussion certainty) x 2(treatment) mixed-model Analysis of Variance (ANOVA) showed that group discussion increased decision certainty (M_{pre}=4.36, SD=0.87 to M_{post}=5.25, SD=0.86), $F(1,36)=27.37$, $p<.001$, $\eta_p^2=.432$), with no effects involving treatment, $F(1,36)=0.11$, $p=.919$ (for Means and Standard Deviations, see S1).

Mood

Both prior to the group's decision-making, and immediately after its completion, participants individually filled out the Positive-Affect Negative-Affect Scale (PANAS; Watson,

Clark & Tellegen, 1988). Participants indicated the extent to which they experienced certain feelings and emotions at that particular moment on 5-point scales (1=*very slightly or not at all;* 5=*extremely*). Emotions included ‘enthusiastic’ and ‘interested’ for the Positive Affect scale and ‘nervous’ and ‘afraid’ for the Negative Affect scale. Reliability was excellent for both the positive ($\alpha_{pre\text{-}task}$=.827, $\alpha_{post\text{-}task}$=.872) and the negative scale ($\alpha_{pre\text{-}task}$=.829, $\alpha_{post\text{-}task}$=.856).

Results of Repeated Measures ANOVA revealed that treatment was unrelated to positive and negative affect before and after the experimental task, all *F*s <.06, *p*s>.488; for Means and Standard Deviations, see Table S2.

Group identification, discussion thoroughness and focus on speed

After the group discussion participants filled out a 14-item questionnaire about their identification with the group (e.g., “I am happy to be a member of my group” α=.925), a 7-item questionnaire on the thoroughness of the group discussion and information processing (e.g., “During the discussion we talked about a lot of information” and “We discussed the information thoroughly”; α=.723); and finally a 6-item questionnaire on the extent the group focused on reaching a decision quickly (e.g., “We tried to reach agreement as quickly as possible”; α=.586). For Means and Standard Deviations of the oxytocin and placebo groups, and tests of significance, see Table S3.

References

Hayes, A. F. (2013). *Introduction to Mediation, Moderation, and Conditional Process Analysis: A Regression-Based Approach*. New York: Guilford Press.

Watson, D., Clark, L. A., & Tellegen, A. (1988). Development and validation of brief measures of positive and negative affect: the PANAS scales. *Journal of Personality and Social Psychology*, *54*(6), 1063–70. http://doi.org/10.1037/0022-3514.54.6.1063

Figures and Tables

	Oxytocin		Placebo		$CI_{95\%}$*
	Mean (*SD*)	*SE*	Mean (*SD*)	*SE*	
**Before group discussion**					
Pre-discussion certainty	4.39 (0.66)	0.15	4.33 (1.07)	0.24	–0.64 0.53
**During group discussion**					
Total information exchange	21.79 (11.54)	2.65	15.95 (10.57)	2.42	–9.89 1.05
Proportion unique information	0.49 (0.18)	0.04	0.34 (0.22)	0.05	–0.28 –0.02
Repetition unique information	2.12 (0.61)	0.14	1.36 (0.97)	0.22	–1.30 –0.23[a]
Repetition shared information	1.47 (0.43)	0.10	1.35 (0.36)	0.08	–0.38 0.14[a]
**After group discussion**					
Post-discussion certainty	5.26 (0.85)	0.20	5.25 (0.89)	0.20	–0.59 0.55
Agreement with group decision	5.96 (0.72)	0.16	6.12 (0.60)	0.14	–0.28 0.59
Ease of group decision	3.11 (1.52)	0.35	3.68 (1.50)	0.34	–0.41 1.57

Table S1

Means, SD’s and SE’s of process measures. *95% Confidence interval of the mean difference.

[a]Ordinal variable, mean differences needs to be interpreted with caution.

	Oxytocin	Placebo	$CI_{95\%}$*
Negative Affect Pre	2.82 (0.56)	2.84 (0.44)	–0.34 0.38
Positive Affect Pre	1.23 (0.23)	1.29 (0.28)	–0.13 0.25
Negative Affect Post	3.02 (0.67)	3.00 (0.59)	–0.44 0.39
Positive Affect Post	1.22 (0.27)	1.35 (0.34)	–0.07 0.33

Table S2.

Means, SD's of the mood measures. *95% Confidence interval of the mean difference.

	Oxytocin	Placebo	t-test	$CI_{95\%}$*
Group Identification	4.17 (1.03)	4.29 (1.15)	*t*(112)=0.64, *p*=.522†	–0.27 0.54
Discussion Thoroughness	5.48 (0.82)	5.13 (0.89)	*t*(36)=–1.70, *p*=.098	–0.76 0.07
Focus on Speed	3.83 (0.94)	4.26 (0.86)	*t*(36)=1.90, *p*=.065	–0.03 0.89

Table S3.Means, SD's and differences between conditions (t-test) of the Group Identification, Discussion Thoroughness, and Focus on Speed measures. All analyses were done with Mixed-Model analyses on the individual level data with a random intercept included to account for group membership. † For this variable, the random intercept was redundant, and this analysis is therefore equal to a t-test on the individual level data. *95% Confidence interval of the mean difference.

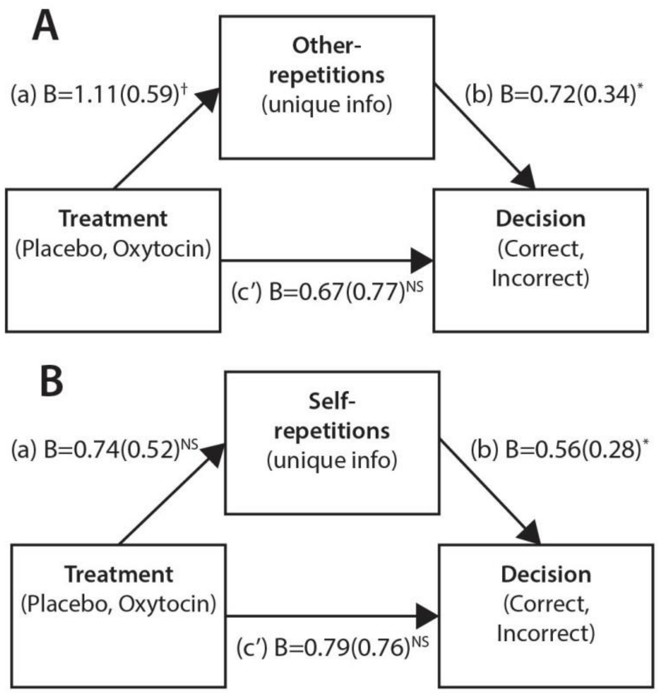

Fig S1.

Mediation Models. Mediation with other-repetition (A). SEs are between parentheses Estimates are based on bootstrapping procedure with 10,000 bootstrap samples. †$p<0.10$;*$p<0.05$. Used Process (Hayes, 2013) Model 4. Path (a) $t(36)=1.87$, $p=.070$; Path (b) $Z(1)=2.09$, $p=.037$; Path (c) $Z(1)=0.87$, $p=.384$. Mediation model using self-repetition (B). SEs are between parentheses Estimates are based on bootstrapping procedure with 10,000 bootstrap samples. *$p<0.05$. Used Process (Hayes, 2013) Model 4. Path (a) $t(36)=1.41$, $p=.17$; Path (b) $Z(1)=2.01$, $p=.044$; Path (c) $Z(1)=1.04$, $p=.301$.
